# Establishment of a Host-to-Host Transmission Model for *Mycobacterium avium* subsp. *hominissuis* Using *Caenorhabditis elegans* and Identification of Colonization-Associated Genes

**DOI:** 10.3389/fcimb.2018.00123

**Published:** 2018-04-24

**Authors:** Luiz E. Bermudez, Sasha J. Rose, Jamie L. Everman, Navid R. Ziaie

**Affiliations:** ^1^Department of Biomedical Sciences, College of Veterinary Medicine, Oregon State University, Corvallis, OR, United States; ^2^Department of Microbiology, College of Science, Oregon State University, Corvallis, OR, United States

**Keywords:** *M. avium*, transmission, host-to-host, colonization genes, *C. elegans*

## Abstract

*Mycobacterium avium* subsp. *hominissuis* (*M. avium*) is a member of the non-tuberculous mycobacteria (NTM), and is a common cause of lung infection in patients with chronic NTM lung conditions. *M. avium* is an environmental bacterium believed to be transmitted from environmental sources. In this work we used a recently developed model in *Caenorhabditis elegans* to ask whether *M. avium* can be transmitted from host-to-host, and the bacterial genes associated with host colonization. Infection of *C. elegans* was carried out by placing the nematode in cultured with *M. avium*. Bacteria eliminated from the intestines of infected *C. elegans* were used to infect naïve nematodes. In parallel experiments, to identify colonization associated genes, a transposon library of *M. avium* was screened for the ability to bind to HEp-2 mucosal cells. Thirty clones were identified and five selected clones with impaired adherence to HEp-2 epithelial cells were used to infect *C. elegans* to determine the degree of colonization. It was determined that *M. avium* eliminated from infected *C. elegans* were able to colonize a naïve *C. elegans* with high efficiency. Thirty of the most adherence-deficient *M. avium* clones obtained from the HEp-2 cell screening were sequenced to identify the location of the transposon. Many of the genes associated with the bacterial cell wall synthesis were shown to be inactivated in the selected mutants. Five out of the 30 bacterial clones were then used to infect *C. elegans*. All five mutants had impaired ability to colonize *C. elegans* compared with the wild type bacteria (decrease of 1.5–2.0 logs, *p* < 0.05). The limitation of this work is that the model can be used for initial screening, but other more complex systems should be used to confirm the findings. *C. elegans* can be used as a model to test for *M. avium* adherence/colonization-associated virulence determinants. All the tested adherence-deficient clones that were examined had impaired ability to colonize the host *C. elegans*, and some can be potentially used to prevent colonization.

## Introduction

Infections caused by non-tuberculous mycobacteria (NTM) are becoming increasingly common worldwide (Primm et al., 2004; Falkinham, 2009). Individuals with chronic lung diseases, such as emphysema, bronchiectasis, and cystic fibrosis have enhanced susceptibility to infections caused by *Mycobacterium avium* subsp. *hominissuis* and *Mycobacterium abscessus* (Griffith et al., 2007; Ringshausen et al., 2016). *M. avium* subsp. *hominissuis*, is the most commonly isolate obtained from patients with lung disease, although the pathogen is also seen associated with disseminated infections in patients with AIDS and other immunosuppressive conditions (Faria et al., 2015; Ringshausen et al., 2016). Options for treatment of NTM infections are limited mainly as a consequence of the intrinsic resistance of this group of microorganisms to the majority of the currently available antibiotics, a fact amplified by the absence of drug discovery programs (Adjemian et al., 2012).

Because NTM are generally environmental microorganisms, it has been assumed that exposure to environmental conditions would be the most common form of transmission to the host (Namkoong et al., 2016). The fact, however, as with many infectious diseases, transmission models are difficult to develop, which creates limitations with the ability to investigate and ultimately understand how pathogens spread among hosts. In addition, to prove that an environmental infection can also be transmitted from host-to-host, and to examine strategies that can be used to prevent infections, is definitively challenging.

Recently, an outbreak of NTM caused by *M. abscessus* in an intensive care unit brought up the importance of the transmission of NTM by other means that differ from the accepted environmental sources (Vaghaiwalla et al., 2014). The numerous or perhaps the prolonged exposure to infected individuals may lead to the transmission of NTMs to other individuals, however, the possibility of developing disease is strongly connected to the immune status of the host and probably the duration of contact (Jamal et al., 2014). Therefore, epidemiologically, it is quite difficult to establish the link between the source of transmission and infection (or disease), with the rare exceptions of the few outbreaks, in which an environment source was suspected, and the connection has been established (Aitken et al., 2012).

We have established the *Caenorhabditis elegans* as an experimental model of *M. avium* subsp. *hominissuis* (*M. avium*) colonization and infection (Everman et al., 2015). It is well-accepted that *M. avium* infects individuals by both respiratory and intestinal routes (Sangari et al., 2000; Babrak et al., 2015). Therefore, the establishment of a transmission model, that can be used to investigate infections acquired from the environment as well as from other hosts, is desirable. Since *M. avium* is a bacterium encountered in many environmental sources sharing common sites with *C. elegans*, we hypothesized that the nematode has the natural possibility of being infected by *M. avium*. Recent results demonstrated that *M. avium* can in fact infect *C. elegans* and invade the intestinal mucosal cells in a similar manner that it does in humans (Sangari et al., 2000; Babrak et al., 2015). The model developed takes advantage of the ability to genetically manipulate *C. elegans* and its environment.

With the establishment of this model, we now can ask questions related to transmission and “colonization” of host by *M. avium*. In this report we show that *M. avium* infection can be transmitted between nematode hosts and identified bacterial genes involved in the successful colonization or attachment to the epithelial mucosa of the new host.

## Materials and methods

### Host cells and *C. elegans*

*C. elegans* strain N2 was maintained in monoxenic cultures with the addition of *Escherichia coli* strain OP50, and propagated on nematode growth medium (NGM) agar plates at 25°C as previously described by Brenner (1974). Human epithelial cells (HEp-2) were obtained from the American Type Culture Collection (ATCC, Manassas, VA, USA). HEp-2 cells were grown in RPMI-1640 medium supplemented with 10% heat-inactivated fetal bovine serum (Gemini Bio-Products, West Sacramento, CA, USA) at 37°C in an atmosphere containing 5% CO_2_.

### Bacteria

All the experiments were carried out under biosafety level 2 containment (BSL2). *E. coli* strain OP50 was grown in Luria Bertani broth overnight, prior to inoculation of NGM plates at 37°C. *M. avium* subsp. *hominissius* strain 104, and *M. avium* subsp. *hominissuis* strain A5 were grown onto Middlebrook 7H10 agar supplemented with 10% w/v oleic acid, albumin, dextrose, and catalase (OADC, Hardy Diagnostics, Santa Maria, CA, USA) for 10 days at 37°C. Bacteria lawn was established on agar plates to feed *C. elegans*. All the studies were carried out in biosafety level 2 laboratory, and the protocols have been reviewed and approved by the Biosafety Committee of the University.

### Construction and screening of a transposon library

*M. avium* mutants were obtained from the screening of a transposon library recently created in our laboratory (Rose and Bermudez, 2016). Briefly, *M. avium* subsp. *hominissuis* strain MAC A5 was transduced with the MycomarT7 phagemid at an MOI of 2 for 4 h at 37°C. Aliquots of the transduction were plated onto Middlebrook 7H10 media containing 400 μg/ml of kanamycin. The library was screened by culturing HEp-2 cells in RPMI-1640 supplemented with 10% heat-inactivated FBS in a 6-well tissue culture plate and adding to it a suspension containing 5,000 pooled clones from the *M. avium* A5 library for 45 min at 37°C. After the 45 min incubation, the supernatant containing bacteria that did not adhere to the cell monolayer, was removed and added to a different monolayer of HEp-2 cells for an additional 45 min. The process was repeated 8 times for enrichment of clones that could not bind to the cells (without passage in culture medium) and the bacterial suspension of the last passage, containing clones that failed to bind to HEp-2 cells, plated onto 7H10 agar. After 10 days, isolated colonies were obtained and tested individually for the ability to bind to HEp-2 cells using the described protocol (Bermudez and Young, 1994). Briefly, individual clones were incubated with 100% confluent HEp-2 cell monolayer for 1 h at 4°C. After the period, the supernatant was removed, the monolayers gently washed once with HBSS, and then the monolayers were lysed as previously described (Bermudez and Young, 1994). The number of adherent bacteria in each clone tested was compared to the adherence of the wildtype bacterium, and the clones which have a 80% or greater decrease in binding to the HEp-2 epithelial cells were selected. Clones impaired in binding were submitted to ligation-mediated PCR and DNA sequencing for identification of the location of the transposon, as previously described (Rose and Bermudez, 2016).

### Transmission assay

The transmission assay was performed by placing *C. elegans*, initially grown on a layer of *E. coli* OP50, on starvation media for 3 days (25°C) and then removing the nematode and placing it on a lawn of *M. avium* A5, *M. avium* 104, or mutants (1 × 10^5^ bacteria) for 4 h (25°C). After the period of time, *C. elegans* were removed out of the plate, the bacteria bound to the outside of the nematode body cleared as previously described (Everman et al., 2015). *C. elegans* (20 worms) were then placed onto an agar plate without bacteria for 2 h at 25°C. In the next step, infected *C. elegans* were then removed from the agar using a platinum wire pick. Fresh, uninfected *C. elegans*, maintained without eating for 3 days, were transposed to the plate that at this point contained bacteria excreted from the previously removed *C. elegans*. After an additional 2 h, the plates were washed in M9 saline (Everman et al., 2015) supplemented with 25 mM of levamisole hydrochloride (Sigma-Aldrich) for paralysis and prevention of elimination or uptake of bacteria during the washes. *C. elegans* were then exposed to amikacin sulfate (200 μg/ml) for 30 min to kill all bacteria bound to the outside of the nematode body (Everman et al., 2015), and subsequently washed twice in HBSS. To quantify bacteria ingested by *C. elegans*, suspensions of 10 nematodes were homogenized using a handheld motorized pot (VWR, Radnor, PA, USA) for 1 min in 0.1% triton X-100. Then, samples were diluted in sterile water, plated onto Middlebrook 7H10 agar, and 10 days later the number of viable bacteria determined. As a control for the bacterial killing in the surface, *C. elegans* were placed and “rolled over” in a LB agar plate as well as a Middlebrook 7H10 agar plate. Plates were checked for mycobacterial growth for 2 months.

### Microscopy (histology and transmission electron microscopy)

For histology worms were collected in M9 saline solution, washed twice in saline with centrifugation at 225 × g for 2 min to remove potential extracellular bacteria. Nematodes were fixed in 10% buffered formula for 5 min (24°C) and placed in melting agarose. Agarose-encased worms were embedded in resin and section mounted into glass slides. Specimens were stained with acid-fast stain and visualized.

### Transmission electron microscopy

Worms collected in M9 solution were washed twice and the pellet was suspended in fixative buffer with 2.5% glutaraldehyde, 1% formaldehyde, and 0.1 M sodium cacodylate. Sections were stained, dehydrated and visualized in the TEM facility of Oregon State University.

### Statistical analysis

Results reported represent the data obtained in at least two experiments performed independently ± standard error. Analysis was done by using Graphpad Prism 6. The statistical significance of the binding assays were determined using the Student's *t*-test and by the ANOVA test. *p* < 0.05 meaning statistical significance.

## Results

### Transmission of *M. avium*

**Figure 1:**
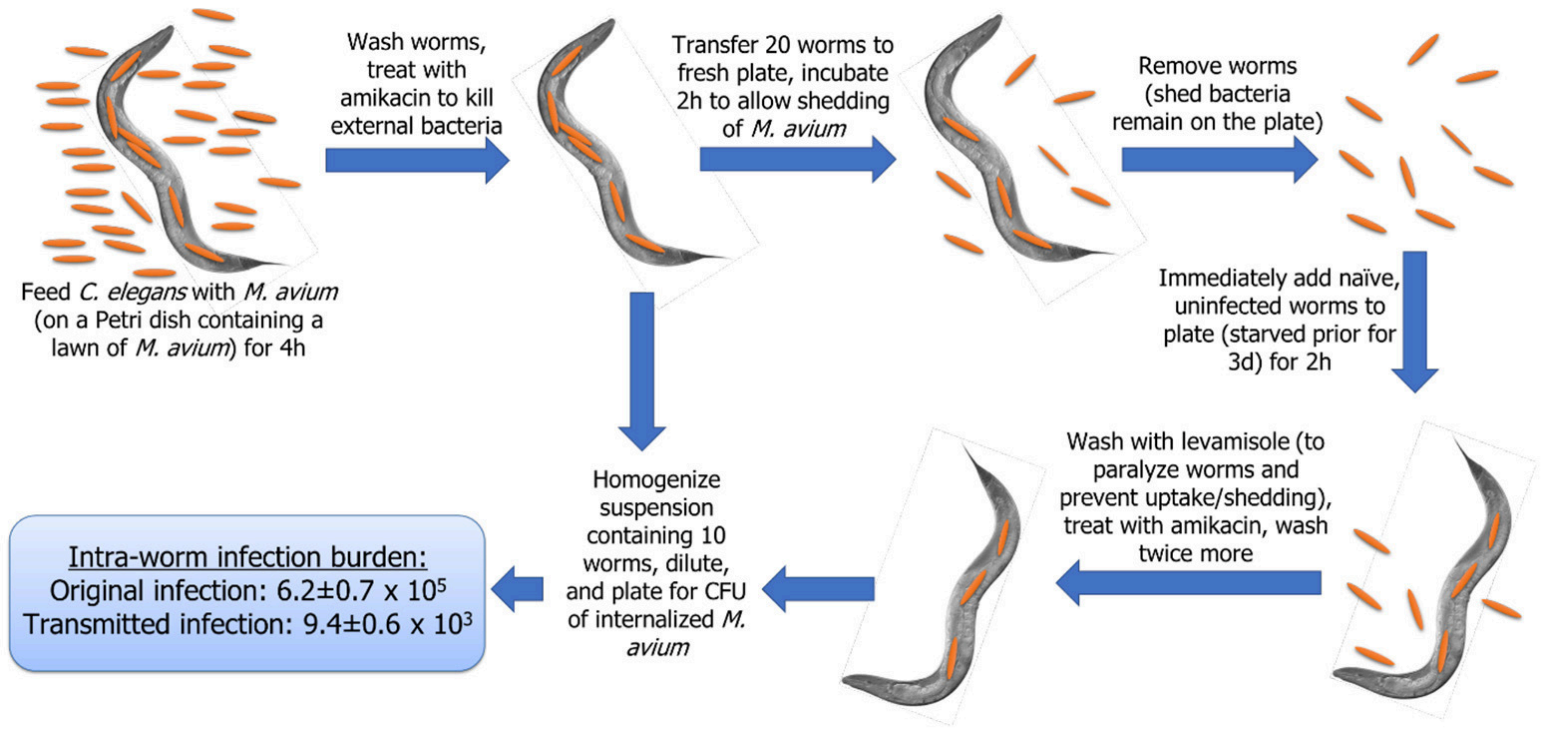
Schematic representation of the model used.

To determine whether *M. avium* could be transmitted from one host (infected) to another (naïve) host without prolonged passage in the environment, *M. avium* strain 104 was used to infect *C. elegans* orally (by seeding it on a plate) and then the nematode was transferred to another plate, this one without a lawn of bacteria, as described in section Materials and Methods and summarized in Figure 1.

**Figure 2:**
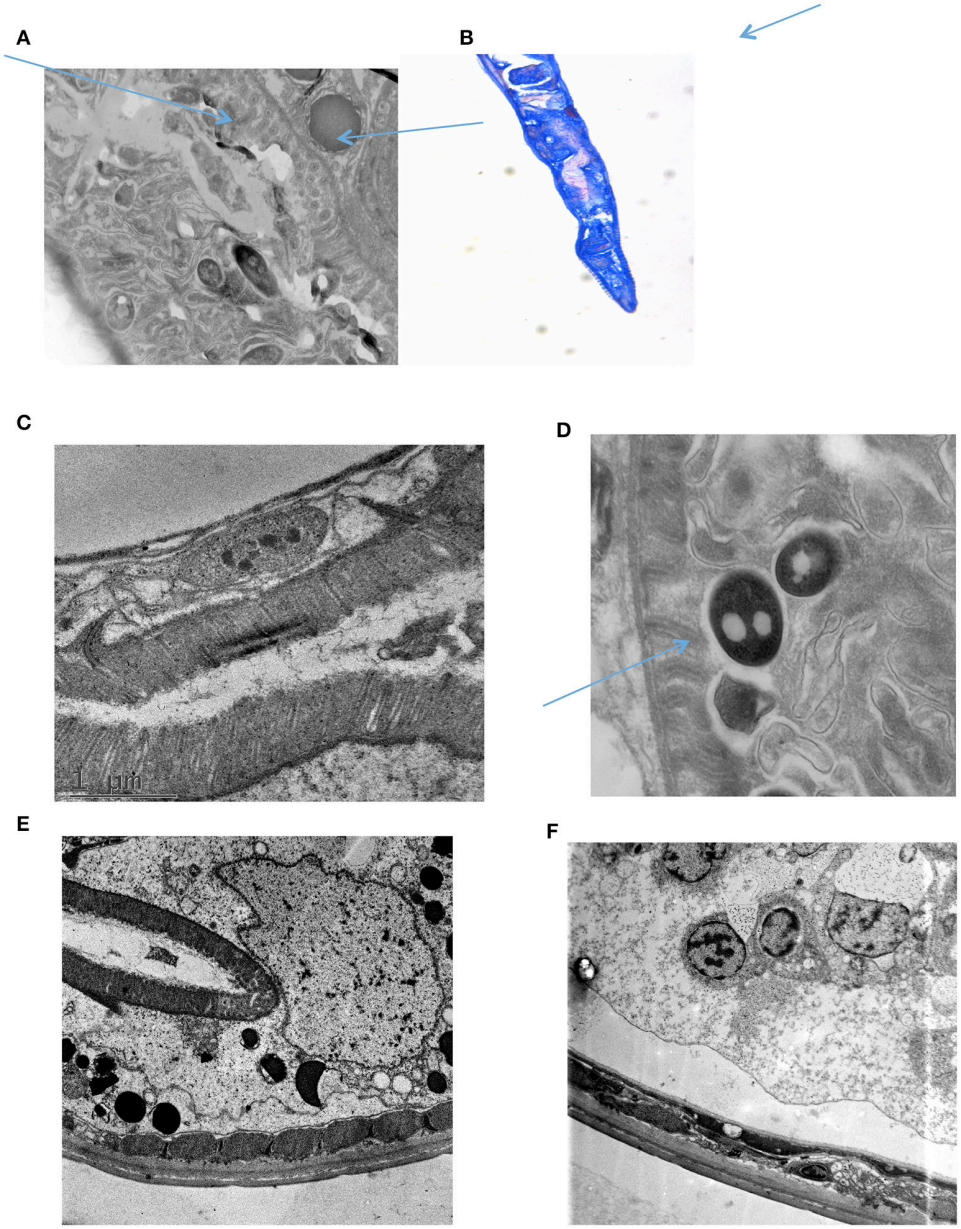
Transmission electro microscopy showing the bacteria interacting with the intestinal mucosa. **(A)** *C. elegans* intestine showing intraluminal bacteria (long arrow) and attached bacteria (short arrow); **(B)** Histopathology of *C. elegans* intestinal tract following MAH infection for 2 h. Arrow area contains many *M. avium* (arrow). **(C)** Control starving *C. elegans*. **(D)** Image showing the binding of *M. avium* with the intestinal mucosa (arrow). **(E)** Mutant E9; **(F)** Mutant H9.

After the elimination of bacteria by the first nematode hosts (infected), the *C. elegans* were again removed from the plate and substituted by uninfected, starving *C. elegans* (naïve). After 2 h, previously uninfected nematodes were removed and their out-surface sterilized. The nematodes were then homogenized and plated for quantification of CFU. Some of the nematodes were prepared for histopathology and electron microscopy as previously described (Everman et al., 2015). As shown in Figures 2A,B, infection was transmitted from one *C. elegans* to another and the pathogen can interact with the intestinal mucosa. The infected *C. elegans* had 6.2 ± 0.4 × 10^5^ bacteria (MAH104) or 3.9 ± 0.5 × 10^4^ A5 strain in the intestines, while the initially naïve nematodes had an average of 9.4 ± 0.6 × 10^3^ (MAH) or 6.7 ± 0.2 × 10^3^ (A5) bacteria in the intestinal lumen after exposed to bacteria (Table 1).

**Table 1:** Transmission of *M. avium* strains 104 and A5 between *C. elegans*.

***M. avium* strain**	**# Bacteria first *C. elegans***	**# Bacteria second *C. elegans***
104	6.2 ± 0.4 × 10^5^	9.4 ± 0.6 × 10^3^
A5	3.9 ± 0.5 × 10^4^	6.7 ± 0.2 × 10^3^

*The numbers represent the average of 2 experiments with 20 C. elegans (first host) and 20 C. elegans (second host) for each of the M. avium strains. The results of two experiments were very comparable*.

### Screening for colonization mutants

It was then decided to select for clones that do not bind human epithelial cells to verify if those clones were also deficient in “colonizing” *C. elegans*. A pooled transposon library of 5,000 mutants was screened for impaired ability to bind to human HEp-2 epithelial cells. After eight serial infections with the same pooled library inoculum that was intended to enrich for clones that were impaired in binding, the final supernatant was removed from HEp-2 cells and plated. Ninety-two colonies from this enrichment were re-grown, and re-tested individually for their binding ability to HEp-2 cells. Forty-seven of the 92 clones showed a greater than 50% reduction in binding to HEp-2 cells compared with the wildtype bacterium. Thirty-seven clones were chosen for sequencing, and in 30 out of 37 clones the gene interrupted by the transposon was successfully identified (Table 2). From the 30 clones sequenced, 29 were unique gene sequences while one gene had a repetitive sequence. This information suggests that although we did not screen the library to saturation (library size of 100,000 clones) we already obtained repetitive genes in the screening, suggesting the gene importance for binding and colonization. Among the genes identified, there were 5 hypothetical proteins (all probable membrane proteins). The ontology of the class of protein encoded genes is show in Table 3. Two genes encoded for membrane proteins with homology to genes in *M. tuberculosis, M. avium*, and *M. abscessus*. Among the identified domains, one (FAM53) is known to bind to eukaryotic cell membrane. A TetR transcription regulator, that likely influences the expression of surface-related proteins, was also identified. Four proteins do not have the function known, and with the exception of one of them (clone G12), which has homology to a flagella protein, do not have known motifs. An oxidoreductase, monooxygenases (2 of them), and aminotransferase were enzymes linked with the phenotype. Out of them, we have evidence for an oxidoreductase from *M. avium* subsp. *paratuberculosis* to be important for the entry of the bacteria in epithelial cells (Alonso-Hearn et al., 2008), by participating on the folding of invasion-related proteins.

**Table 2:** Clones deficient in rapid attachment to HEp-2 cells.

**Clone**	**Reduction from WT (%)^a^**	**Gene**	**Encoded protein**	**Homologs^b^/location Tn^c^**
E4	98.2	MAVA5_06540	Dihydropteroate synthase	MAV_1352, Rv1207, MAB_1345/58 n C-terminus
D6	95.4	MAVA5_10295	4-hydroxyacetophenone monooxygenase	MAV_1795, MAB_4476c 104 n N-terminus
D3	93.8	MAVA5_03280	Mycothiol acetyltransferase	MAV_0761, Rv0819, MAB_0748/112 n N-terminus
H8	93.7	MAVA5_14105	Methylmalonyl-CoA mutase	MAV_3277, Rv1493, MAB_2711c/110 n N-terminus
H4	92.2	MAVA5_09730	Hydrolase	MAV_2243, Rv2223c, MAB_1919/106 n N-terminus
E6	91.5	MAVA5_15805	Acyl-CoA dehydrogenase	MAV_3616, Rv2724c, MAB_3040c/120 n N-terminus
G5	90.1	MAVA5_18755	Serine/threonine protein kinase	MAV_4238, Rv0931c/96 n C-terminus
G10	89.3	MAVA5_04860	Major facilitator transporter	MAV_1023, Rv2456c, MAB_3449c/146 n N-terminus
D10	88.3	MAVA5_03005	Alcohol dehydrogenase	MAV_0705, Rv0761c, MAB_4560/ middle of gene
H3	88.0	MAVA5_15805	Acyl-CoA dehydrogenase	MAV_3616, Rv2724c, MAB_3040c/ 101 n N-terminus
D7	87.9	MAVA5_15910	N-acetylglutamate synthase	MAV_3638, Rv2747, MAB_3072/ 106 n N-terminus
D8	86.6	MAVA5_21725	Succinate-semialdehyde dehydrogenase	MAV_4936, Rv0234c, MAB_3471/ 121 n N-terminus
D5	79.8	MAVA5_10295	4-hydroxyacetophenone monooxygenase	MAV_1795, MAB_4476c/ 105 n N-terminus
C12	79.5	MAVA5_14485	Cupin	MAV_3361/ 66 n C-terminus
H11	79.5	MAVA5_22225	TetR family transcriptional regulator	MAV_5138, Rv0158, MAB_4574c/ 104 n N-terminus
F10	79.3	MAVA5_17170	4-hydroxyacetophenone monooxygenase	MAV_3915, Rv3049c, MAB_3920c/ 106 n N-terminus
H5	78.9	MAVA5_10525	Carboxylate-amine ligase	MAV_2378, Rv2125, MAB_2128c/ 109 n N-terminus
B5	76.2	MAVA5_11640	Oxidoreductase	MAV_2766/ Middle of gene
H9	73.7	MAVA5_08165	Membrane protein	MAV_1726, Rv2446c, MAB_1605c/ middle of gene
F12	73.6	MAVA5_12410	Hypothetical protein	MAV_2925, Rv1787/ middle of gene
E9	65.8	MAVA5_01195	Aminotransferase	MAV_0250, Rv3772, MAB_0220/ 210 n N-terminus
E2	64.6	MAVA5_08520	Acetyl hydrolase	MAV_1798, Rv2385, MAB_2076/121 n N-terminus
E12	63.0	MAVA5_11775	Hypothetical protein	None/96 n N-terminus
G12	59.7	MAVA5_15020	Hypothetical protein	MAV_3472/111 n N-terminus
B2	58.4	MAVA5_06340	Hypothetical protein	MAV_1314, Rv1174c, MAB_2488c/middle of gene
F9	54.5	MAVA5_04920	Hypothetical protein	None/105 n N-terminal
C2	54.4	MAVA5_22565	Monooxygenase	MAV_5206/42 n C-terminus
C5	54.2	MAVA5_02065	Inhibition of morphological differentiation protein	MAV_0469, Rv3661, MAB_0431c/110 n N-terminus
C9	53.1	MAVA5_02460	ABC transporter ATP-binding protein	None/32 n C-terminus
A7	52.9	MAVA5_10715	Hypothetical protein	None/middle of gene

a *This value was calculated from the CFU recovered bound to and/or invaded in the epithelial cells between the average of four wildtype samples with the respective clone*.

b *The respective clone was compared with the type strains M. avium subsp. hominissuis 104, M. tuberculosis H37Rv, and M. abscessus subsp. abscessus 19977 to determine homology*.

c *Location of transposon: Nucluotides from C- or N-terminus*.

**Table 3:** Ontology of the 30 genes sequenced that were associated with binding and/or colonization of the epithelial mucosa.

**Pathways/groups of genes**	**Number of genes associated with phenotype**
Metabolic pathways	8
Signaling	1
Transport Proteins	2
Transcription regulators	1
Membrane proteins	5
Folate, DNA synthesis	1
Flavoprotein, energy	1
Fatty acid synthesis	6

The clone B2 has MAVA5_06340 gene interrupted. Sequence information tells that the gene exists in both A5 and 104 strains as an isolated gene. The E9 clone (MAV_01195) is also an isolated gene. The clone G12 (MAV_15020) is the gene in the end of a two-gene operon. The clones H9 and B5, the genes interrupted are in the middle of the operon, what require complementation to rule out the phenotype being due to a downstream gene.

### Evaluation of the *M. avium* mutants for colonization of *C. elegans*

To determine if the clones with impaired ability to bind to human mucosal epithelial cells were also deficient in transmission between nematodes, we infected *C. elegans* orally with each, the wildtype bacteria and the binding-deficient clones. We also determined the growth of bacterial clones on Middlebrook 7H9 and 7H10 media. All of the mutants used (B5, E2, H9, G12, and E9) grew in a comparable fashion to the wildtype bacterium (MACA5).

As shown in Figures 2A–D, *M. avium* can infect *C. elegans* with attachment and invasion of the intestinal tract mucosa. Based on that information, we selected 5 mutants that showed impairment to adhere to human epithelial cells *in vitro*, and one positive control mutant (4B2, deficient in GPL) that does not adhere to epithelial cells efficiently. The results obtained in *C. elegans* confirmed the deficiency observed of the clones to attach to human epithelial cells (Table 4). All five tested clones showed impairment of infection that were statistically significant.

**Table 4:** Binding of wild type and mutant strains to *C. elegans* intestine.

**Bacteria strain/clone**	**CFU/plate**	**CFU/worm intestine/ 30 min^a^**	***P*-value^b^**
MAH 104	6.4 × 10^5^	4.7 ± 0.6 × 10^4^	–
MAH A5	5.9 × 10^5^	3.0 ± 0.5 × 10^4^	–
B5	5.6 × 10^5^	2.2 ± 0.4 × 10^3^	<0.05
B2	6.4 × 10^5^	5.7 ± 0.4 × 10^2^	<0.02
H9	5.2 × 10^5^	2.1 ± 0.4 × 10^2^	<0.01
G12	5.3 × 10^5^	2.7 ± 0.6 × 10^2^	<0.02
E9	6.1 × 10^5^	1.5 ± 0.8 × 10^2^	<0.01
4B2 (ΔGPL)	5.9 × 10^5^	1.4 ± 0.3 × 10^3^	<0.05

a *10 C. elegans were exposed to MAH/excretion of 20 C. elegans*.

b *The calculation of statistical significance was carried out using the ANOVA test. P <0.05 was considered significant*.

## Discussion

*M. avium* infection of the lung is believed to be always acquired from an environmental source (Griffith et al., 2007; Adjemian et al., 2012; Ringshausen et al., 2016). Recent events suggested that it may not be the case (Vaghaiwalla et al., 2014), and environmental mycobacteria could be transmitted from host-to-host in unidentified occasions. Assuming that it was the case, what makes it difficult to establish the epidemiologic link is that the bacterium could in fact infect the host weeks or even months before the clinical disease would develop and could be diagnosed and linked to the source.

In this work we used the nematode *C. elegans* to establish a transmission model, in which an *M. avium* strain eliminated from one *C. elegans* can be transmitted, without remaining in the environment for an extended period of time, to a “naïve” *C. elegans* and ultimately establish infection. Once it was demonstrated that the *C. elegans* model was feasible to study transmission, we screened a transposon library of *M. avium* for clones that had deficiency in attaching to human mucosal epithelial cells, and then used the selected mutants in the *C. elegans* assay. All the five mutants selected were shown to have impaired ability to bind to the intestinal mucosa of *C. elegans*.

The initial indication is that the screening *in vitro* for deficiency in binding to epithelial cells seems to provide results that can be reproduced in other models. In despite of the fact that we only examined five adherence-deficient out of 30 clones using the *C. elegans* model, the results obtained suggest that the system can be employed to study transmission and “colonization” and perhaps, in the future, how to prevent it.

The second important finding is that the model confirmed the possibility that in many occasions *M. avium* may be acquired from a living source, such as an infected patient with chronic pulmonary condition, instead of from the outside environment. Expending the model to determine the virulence determinants in the pathogen which are associated with infection can be advantageous over the current available systems. Furthermore, *C. elegans* is a genetically treatable host, and mutations can be obtained in the genes with potential to be associated with mechanisms of host innate defense.

The final finding of this work is that genes were identified in association to binding, and, when inactivated, resulted in significant decrease of adherence to human respiratory epithelial cells as well as the ability of the mutant clone to colonize *C. elegans*. A number of the identified genes encode for proteins of unknown function, while other genes identified encode for enzymes involved in the synthesis of cell wall or surface structures, fatty acid synthesis, metabolic pathways, and transporters among others (Tables 2, 3). The latter may be related with the assemble of the outer surface of the bacterium. The consequent conclusion is that the pathogen seems to utilize several molecules to interact with the surface of the epithelial cells. It would be important to determine if there is a hierarchy of molecules that follow the attachment or whether the process is indiscriminate or partially redundant. That information may have important consequences to the development of a strategy to diminish or prevent colonization.

The limitations of the current study is that *C. elegans* system appears to work as initial screening, but the phenotype should be confirmed in a more complex system, as done with the strain 4B2.

In summary, we established a model of host-to-host transmission of NTM (*M. avium*), and identified genes associated with attachment of the bacterium to the host mucosa. Future work will explore the model in the attempt to identify strategies to prevent colonization.

## Author contributions

LB performed experiments, wrote the paper, principal investigator. SR performed experiments, wrote the paper. JE and NZ perform experiments.

### Conflict of interest statement

The authors declare that the research was conducted in the absence of any commercial or financial relationships that could be construed as a potential conflict of interest.
